# Comparative analysis of enoxaparin versus rivaroxaban in the treatment of cancer associated venous thromboembolism: experience from a tertiary care cancer centre

**DOI:** 10.1186/s12959-020-00221-2

**Published:** 2020-05-24

**Authors:** Anadil Faqah, Hassan Sheikh, Muhammad Abu Bakar, Fatima Tayyaab, Sahrish Khawaja

**Affiliations:** 1 Department of Internal Medicine, Shaukat Khanam Memorial Cancer Hospital & Research Centre, Lahore, Pakistan; 2 Department of Hematology and Oncology, Shaukat Khanam Memorial Cancer Hospital & Research Centre, Lahore, Pakistan; 3 Department of Cancer Registry, Shaukat Khanam Memorial Cancer Hospital & Research Centre, Lahore, Pakistan

**Keywords:** Anticoagulants, Rivaroxaban, Factor Xa inhibitor, Thrombosis, Low-molecular-weight-heparin, Cancer-associated-thrombosis

## Abstract

**Background:**

Venous Thromboembolism (VTE) in cancer patients is associated with increased mortality and morbidity. While newer data on use of direct oral anticoagulants (DOACs) in treating cancer associated thrombosis (CAT) is promising; its data is still few and inconsistent across literature. We designed the study to assess if rivaroxaban would be an appealing alternate choice to treat CAT.

**Methods:**

We conducted a retrospective study to evaluate the efficacy and safety profile of rivaroxaban versus enoxaparin in cancer patients after developing a symptomatic deep vein thrombosis (DVT) or pulmonary embolism (PE). Baseline patient characteristics and laboratory values were assessed in each arm. Primary efficacy outcome was measured by radiographically confirmed VTE recurrence at different intervals. Primary safety outcome was measured by presence of major and minor bleeding using the ISTH scale.

**Results:**

Our study recruited 150 cancer patients with radiologically confirmed DVT and PE; 80 patients were evaluated in enoxaparin arm and 70 patients in rivaroxaban arm. Our results showed that there was no statistically significant difference between the incidence of VTE recurrence at 6 months between the enoxaparin and rivaroxaban arm (10% vs 14.2%, *p* = 0.42). Historically significant risk factors for VTE in cancer patients such as high platelet count, high leukocyte count, low hemoglobin level, high risk gastrointestinal, genitourinary and lung cancers were not found to be significantly associated with the risk of VTE recurrence. Primary safety outcome analysis also showed no statistically significant difference in major (11.2% vs 11.4%) and minor (15% vs 10%) bleeding between enoxaparin versus rivaroxaban arm respectively (*p* = 0.65).

**Conclusion:**

We conclude that there was no significant difference seen between the efficacy and safety profile of enoxaparin and rivaroxaban in our cancer patient population.

## Introduction

Venous Thromboembolism (VTE) which broadly consists of deep vein thrombosis (DVT) and pulmonary embolism (PE) is associated with a poor prognosis in patients with cancer and remains a leading cause of mortality and morbidity [1]. Cancer patients are at 6 to 7 fold increased risk of venous thromboembolism (VTE) compared with age-matched controls corresponding to an annual incidence of about one thrombotic event per 200 active cancer patients [2]. Therefore adequate management of VTE is of utmost importance for clinicians involved in the care of cancer patients.

There has been substantial advances in the management of cancer associated thrombosis (CAT) in the last few decades. Low molecular weight heparin (LMWH) which was once considered the gold standard is no more the only treatment option available [3–5]. Direct oral anticoagulants (DOACs) i.e. rivaroxaban, apixaban, and edoxaban which are taken orally and do not require laboratory monitoring have become an appealing alternate choice as oppose to LMWH which require daily subcutaneous injections. The initial literature on use of DOACs was drawn from meta-analysis evaluating randomized controlled trials (RCTs) with cancer subgroups i.e. RECOVER, AMPLIFY, Hokusai-VTE, EINSTEIN-PE & DVT. They drew conclusion that DOACs were non-inferior to LMWH in preventing recurrent VTE and are associated with similar bleeding rates [6–11]. On the contrary its key criticism stems from the fact that only less than 7% of the study population in these RCTs had cancer.

More recently two randomized control trials (SELECT D & Hokusai VTE- Cancer) have emerged involving the use of DOACs versus LMWH in preventing cancer associated thrombosis [12, 13].These studies showed that DOACs were noninferior to LMWH in preventing recurrent VTE; however this is with increased risk of bleeding. In the randomized SELECT D trial, 203 patients were compared with dalteparin versus rivaroxaban. The VTE recurrence rate for dalteparin versus rivaroxban was 11% versus 4% respectively [HR 0.43 (0.19–0.9)]. However major bleeding risk for dalterparin versus rivaroxaban was 4% versus 6% respectively [HR 1.83 (068–4.96)]. In the randomized Hokusai VTE trial, 1050 patients were compared with LMWH for 5 days followed by oral edoxaban versus dalteparin. The VTE recurrence rate for dalteparin versus edoxaban was 11.3% versus 7.9% respectively. However major bleeding risk for dalterparin versus edoxaban was 4% versus 6.9% respectively.

Following these recent trials, American Society of Clinical Oncology (ASCO) and National Comprehensive Cancer Network (NCCN) have revised their recommendations and have added the use of rivaroxaban and edoxaban for cancer associated thrombosis treatment [14, 15]. Although the recommendations for the use of DOACs have recently become popular in guidelines, they are still few and inconsistent across the current literature. In the absence of multiple large randomized controlled trials and dearth of literature in cancer population we designed a retrospective single center study to investigate the efficacy and safety profile of rivaroxaban over enoxaparin in preventing recurrent cancer associated thrombosis.

## Patients and methods

### Design

This study was a single center retrospective chart review study utilizing data from the Shaukat Khanum Cancer Memorial Hospital and Research Centre (SKMCH) cancer registry between January 1, 2012 to Dec 31,2017 following the approval by the Institutional Review Board. Patients who received anticoagulation therapy with enoxaparin or rivaroxaban from January 1, 2012 to Dec 31,2017 were identified using a report generated from pharmacy charge codes.

### Patient population

Patients were included if they were at least 18 years of age, had a diagnosis of cancer and concurrent radiological diagnosis of DVT and/or PE, and were prescribed treatment with either rivaroxaban or enoxaparin during the study period. Patients were excluded if the length of anticoagulation therapy was less than 30 days, if therapy with enoxaparin or rivaroxaban was initiated more than 6 months after DVT or PE diagnosis, or if they did not receive therapeutic doses of the therapy. Patients with DVT of upper extremity were also excluded.

### Outcome

The primary efficacy outcome was the incidence of radiologically confirmed new or recurrent DVT or PE in 30 days, 3 months and 6 months using fisher exact test. The secondary endpoint of the study was to compare the safety of enoxaparin vs rivaroxaban in cancer patients for the treatment of DVT or PE.

The primary safety outcome was determined by the incidence and severity of bleeding, based on the International Society of Thrombosis and Hemostasis (ISTH) definition. Major bleeding was defined as clinically overt if it was associated with a drop in hemoglobin of 2 g/dL, required transfusions of 2 units of packed red blood cells, involved critical site bleeding (intracranial, intraspinal, intraocular, retroperitoneal, or pericardial area), or if it contributed to death. Minor bleeding was defined as overt bleeding not meeting the criteria for major bleeding but associated with medical intervention, unscheduled contact with a physician, interruption or discontinuation of anticoagulation treatment, or associated with any discomfort or impairment of activities of daily life.

### Study procedure

The following information was extracted from the medical records for each eligible patient: age, gender, demographics, Body-Mass Index, laboratory results at time of VTE diagnosis, cancer type, presence of active cancer, chemotherapy history, metastatic malignancy, comorbidities (coronary artery disease, hypertension, diabetes, renal insufficiency) prior history of VTE, surgery within 30 days or central venous catheter.

Patients who received therapeutic doses of Enoxaparin were matched with a similar population of patients who were treated with therapeutic doses of Rivaroxaban in a 1:1 ratio. Wilcox in rank sum test was performed to compare continuous variables. The Fisher exact test was performed to compare categorical variables. All data were analyzed using SAS 9.4 with a significance level of a = 0.05.

## Results

### Patient population

**Fig. 1:**
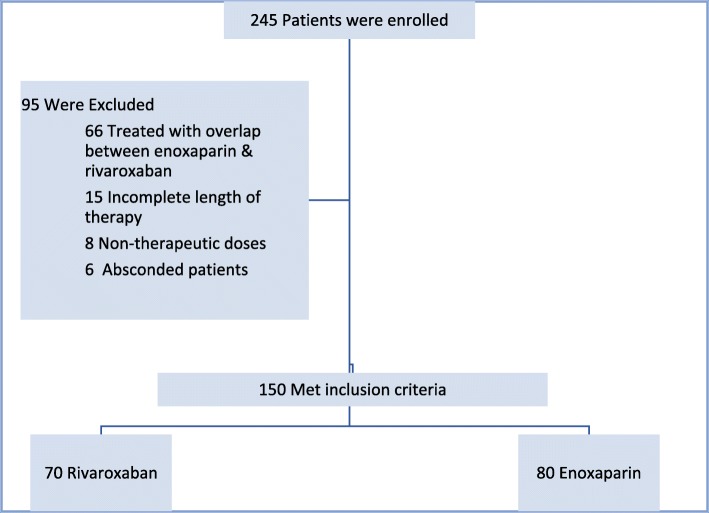
Enrollment Flow Chart

Between January 12,012 to December 31, 2017, a total of 245 patients were screened and 150 eligible patients were included in the study; 95 patients excluded from the study consisted of those who had treatment for less than 6 months, administered non-therapeutic doses, absconded and treatment overlap with both rivaroxaban and enoxaparin (Fig. 1). Of the total 150 patients, 80 patients were treated with enoxaparin and 70 patients were treated with rivaroxaban.

The baseline characteristics, comorbidities were reasonably comparable between treatment arms. (Table 1) except for average leukocyte count 9.67+/− 4.96 in the enoxaparin arm compared to 8.16 +/− 3.79 in rivaroxaban arm; also average albumin level was 3.46+/− 0.78 in enoxaparin arm compared to 3.75+/− 0.56 in rivaroxaban arm. Indication for anticoagulation for our population included DVT only 70, PE only 76 and DVT/PE both 4. Interestingly enough baseline comorbidities which included coronary heart disease, diabetes, hypertension and renal insufficiency were similar in both arms and mostly absent. However most of our cohort had active malignancy at the time of VTE diagnosis 90.1%, including 48.05% patients with metastatic disease and 63.4% receiving chemotherapy. GI malignancy was the primary in enoxaparin arm whereas GU malignancy was the primary in rivaroxaban arm. Risk factors for thrombosis such as central line and immobilization/ major surgery were similar in both groups.

**Table 1:** Demographic Table: This will include all the following information in both Enoxaparin and Rivaroxaban Arm

Variables	Enoxaparin 80 (53.3%)	Rivaroxaban 70 (46.7%)	*p*-value
**Baseline Demographics**			
**Age in years**	48.67 ± 14.45	51.84 ± 13.49	0.17
**Gender**			0.25
**Male**	37 (46.2%)	39 (55.7%)	
**Female**	43 (53.8%)	31 (44.3%)	
**BMI**	23.63 ± 4.64	25.20 ± 5.43	0.05
**Baseline Co-Morbids**			
**Coronary Artery Disease**			1.00
**No**	79 (98.8%)	69 (98.6%)	
**Yes**	1 (1.2%)	1 (1.4%)	
**Hypertension**			0.30
**No**	70 (87.5%)	57 (81.4%)	
**Yes**	10 (12.5%)	13 (18.6%)	
**Diabetes Mellitus**			0.49
**No**	64 (80.0%)	59 (84.3%)	
**Yes**	16 (20.0%)	11 (15.7%)	
**Creatinine Clearance**			0.78
**Less than 60**	8 (10.0%)	8 (11.4%)	
**Equal or above 60**	72 (90.0%)	62 (88.6%)	
**Baseline Malignancy History**			
**Active Cancer**			0.58
**No**	9 (11.2%)	6 (8.6%)	
**Yes**	71 (88.8%)	64 (91.4%)	
**Cancer Type**			0.07
**GI**	23 (28.8%)	18 (26.1%)	
**Breast**	9 (11.2%)	17 (24.6%)	
**GU**	21 (26.2%)	22 (31.9%)	
**Lungs**	4 (5.0%)	3 (4.3%)	
**Misc.**	23 (28.8%)	9 (13.0%)	
**Disease status**			0.89
**Non-metastatic**	42 (52.5%)	36 (51.4%)	
**Metastatic**	38 (47.5%)	34 (48.6%)	
**Chemotherapy**			0.82
**No**	30 (37.5%)	25 (35.7%)	
**Yes**	50 (62.5%)	45 (64.3%)	
**Baseline Laboratory Findings**			
**Hemoglobin Level**	10.65 ± 2.19	13.12 ± 14.21	0.13
**Platelet Level**	274.93 ± 140.70	302.60 ± 153.84	0.25
**Leukocyte Count**	9.67 ± 4.96	8.16 ± 3.79	0.04
**Albumin**	3.46 ± 0.78	3.75 ± 0.56	0.01
**Creatinine**	0.81 ± 0.64	0.73 ± 0.27	0.34
**Creatinine clearance**	128.02 ± 82.76	114.30 ± 45.65	0.22

### Recurrent VTE

Table 2 shows a comparison of the incidence of VTE recurrence and bleeding events. Overall, rivaroxaban had a similar rate of VTE recurrence at 6 months with 10 (14.3%) events versus 8 (10.1%) events with Enoxaparin (*p* = 0.42). The incidence of recurrent DVT at 6 months in patients treated with enoxaparin (3.75%) was lower compared to rivaroxaban (8.75%) at 6 months, however there was no statistical significance (*p* = 0.11). The incidence of recurrent PE in patients treated with enoxaparin (6.25%) was higher compared to rivaroxaban with (5.71%) at 6 months, however there was no statistical significance (*p* = 0.08).

**Table 2:** Efficacy and Safety Outcome

Variables	Enoxaparin 80 (53.3%)	Rivaroxaban 70 (46.7%)	*p*-value
Primary Efficacy Outcomes			
VTE Recurrence			0.42
No	72 (90.0%)	60 (85.7%)	
Yes	8 (10.0%)	10 (14.3%)	
• DVT recurrence			0.11
Proximal	3 (100.0%)	6 (100.0%)	
Distal	0 (0.0%)	0 (0.0%)	
• PE recurrence			0.07
Central	5 (100.0%)	2 (50.0%)	
Sub segmental	0 (0.0%)	2 (50.0%)	
Primary Safety Outcomes			
Safety outcome			0.65
No bleeding	59 (73.8%)	55 (78.6%)	
Minor bleeding	12 (15.0%)	7 (10.0%)	
Major bleeding	9 (11.2%)	8 (11.4%)	

### Bleeding

Nine patients receiving enoxaparin had major bleeds, compared with eight patients in the rivaroxaban arm. (Table 2). The cumulative major bleed rate at 6 months was comparable with no significant difference between 11.2% for enoxaparin arm and 11.4% for rivaroxaban arm. An additional twelve patients receiving enoxaparin had minor bleeds, compared with seven patients in the rivaroxaban arm. (Table 2). The cumulative minor bleed rate at 6 months was again comparable with no significant difference between 15% for enoxaparin arm and 10% for rivaroxaban arm.

## Discussion

One of the population based study from Walker European Journal has shown a steady increase in the absolute rate of venous thrombosis from 10 VTE (per 1000 person-years) to 20 VTE (per 1000 person-years) from 1997 to 2007 in cancer patients; where as it has remained steady i.e. 4 VTE (per 1000 person-years) in non-cancer group [1]. This rise in cancer associated thrombosis poses a serious problem that diminishes the patient’s life span and quality of life. Hence identifying adequate management of cancer associated thrombosis is imperative especially when use of anticoagulation is complicated by a delicate balance between risk of recurrent VTE and major bleeding.

Our study showed the cumulative VTE recurrence risk in enoxaparin and rivaroxaban at 6 months is consistent with the current literature. However there was a non-significant increase in VTE recurrence in rivaroxaban as compared to enoxaparin. It was noted that while recurrent VTE estimate in enoxaparin arm was comparable with previous RCTs (HOKUSAI-VTE, HOKUSAI-VTE CANCER and SELECT D); there was higher recurrent VTE estimate in rivaroxaban arm when compared to current data. On analyzing our data we found out that 50.6% of our population indication for anticoagulation was PE while it was only 29.7% in Chaudhury et al. and 39% in Young et al.; explaining that our patient population was more at risk at baseline [16]. We also noted that our study had larger percentage of gastric (7.3% Vs 3%) and cervical cancer (6.6% Vs 3%); which studies have shown to also cause a higher rate of VTE recurrence [17]. We also separately analyzed the demographic details of each one of the 6 recurrent DVT patients in rivaroxaban arm and found that 4 of 6 had gastric or pancreatic cancer, low albumin, BMI less than 22 and shared co-morbid (Diabetes, Hypertension and Coronary Artery Disease). All of which explains low nutritional reserve resulting in poor gut absorption for oral anticoagulant and inconversely higher adverse outcomes. We also analyzed historically significant risk factors for VTE in cancer patients such as high platelet count, high leukocyte count, low hemoglobin level, high risk gastrointestinal, genitourinary and lung cancers. They were not found to be independently significantly associated with the risk of VTE recurrence [18].

Another important objective of our study was to evaluate the safety outcome i.e. to access rates of major bleeding and clinically relevant non-major bleeding (CRNMB). Our study showed comparable major bleeding and CRNMB rates in rivaroxaban and enoxaparin arm as shown in RECOVER/RECOVER-2. It is worth mentioning that the trends in bleeding rate are not consistent across trials [6, 19]. The bleeding rates in our study were strikingly higher (11% Vs 4–7%) when compared with previously observed rates and were mainly related to GI bleed. Although bleeding rates as high as 16% in enoxaparin plus warfarin arm in CANTHANOX 2002 study and 9% in enoxaparin in ONCENOX 2006 study were seen in prior studies; however, no study has shown bleeding rates higher than 7% (HOKUSAI-VTE Cancer) with DOACs.

Rivaroxaban is a factor Xa inhibitor which directly and reversibly binds to factor Xa and competitively inhibits factor Xa. It is 10,000 fold more selective for factor Xa and it does not require co-factors to exert its anticoagulant effect [20, 21]. It is plausible that the enhanced antithrombotic effects of rivaroxaban as opposed to LMWH which acts on factor X indirectly, is associated with a greater perturbation of coagulation and predisposing to more bleeding. We take this as a learning opportunity to consider modifying DOAC doses to best suit your patient’s needs depending on their demographic and disease specific details. When we individually analyzed 8 episodes of major bleeding with rivaroxaban in our study population we found out they were mainly in older population i.e. aged 65 years or higher with poor nutritional reserve i.e. low albumin, BMI less than 22 and had an advanced metastatic breast or prostate cancer.

Our study despite being a retrospective study and having limited number of participants provides solutions for real world situations. It is felt that despite availability of results from SELECT D & Hokusai VTE-Cancer trial our study still manages to highlight that “one size fits all” cannot be applied to all patients and physicians will need to use their best clinical judgement. We believe NOACs have a promising future in cancer associated VTE due to its ease in utilization and comparable results with LMWH in preventing recurrent VTE. However we are still concerned about its safety profile. Especially as our study showed higher rates of major bleeding with Rivaroxaban when compared to SELECT D & Hokusai VTE-Cancer trial. This is particularly true for complex cancer patients due to rivaroxaban’s unpredictable higher risk of GI bleeding, inability to measure anticoagulant activity by using standard essays, potential interaction with medicines and altered metabolism in renal dysfunction, hepatic metastasis and lack of antidote. In addition patients with gastric and pancreatic cancer who have undergone surgical resection will have altered gut absorption hence making rivaroxaban pharmacodynamics even more unpredictable.

## Conclusion

We conclude that there was no significant difference seen between the efficacy and safety profile of enoxaparin and rivaroxaban in our cancer patient population. While rivaroxaban has recently become popular in cancer associated VTE due to its ease in utilization and comparable results with LMWH in preventing recurrent VTE. Attention also needs to be paid on patient’s disease specific details and demographics before favoring DOACs over LMWH.

## Data Availability

The datasets used and/or analysed during the current study are available from the corresponding author on reasonable request.

## Abbreviations

VTE: Venous Thromboembolism
CAT: Cancer Associated Venous Thrombosis
ISTH: International Society of Thrombosis and Hemostasis
LMWH: Low Molecular Weight Heparin
NCCN: National Comprehensive Cancer Network
ASCO: American Society of Clinical Oncology
DOACs: Direct oral anticoagulants
CRNMB: Clinically relevant non-major bleeding
FDA: Food and Drug Association
PE: Pulmonary Embolism
DVT: Deep Vein Thrombosis

## References

[1] Timp JF, Braekkan SK, Versteeg HH (2013). Epidemiology of cancer-associated venous thrombosis. Blood.

[2] Martinez BK, Sheth J, Patel N (2018). Systematic review and meta-analysis of real-world studies evaluating rivaroxaban for Cancer-associated venous thrombosis. Pharmacotherapy.

[3] Lee AYY, Levine MN, Baker RI (2003). Low-molecular-weight heparin versus a coumarin for the prevention of recurrent venous thromboembolism in patients with cancer. N Engl J Med.

[4] Kuderer NM, Lyman GH (2014). Guidelines for treatment and prevention of venous thromboembolism among patients with cancer. Thromb Res.

[5] Streiff Michael B. (2009). An Overview of the NCCN and ASCO Guidelines on Cancer-Associated Venous Thromboembolism. Cancer Investigation.

[6] Schulman S, Kearon C, Kakkar AK (2009). Dabigatran versus warfarin in the treatment of acute venous thromboembolism. N Engl J Med.

[7] Agnelli Giancarlo, Buller Harry R., Cohen Alexander, Curto Madelyn, Gallus Alexander S., Johnson Margot, Masiukiewicz Urszula, Pak Raphael, Thompson John, Raskob Gary E., Weitz Jeffrey I. (2013). Oral Apixaban for the Treatment of Acute Venous Thromboembolism. New England Journal of Medicine.

[8] Büller HR, Décousus H, Grosso MA (2013). Edoxaban versus warfarin for the treatment of symptomatic venous thromboembolism. N Engl J Med.

[9] Landman GW, Gans ROB. Oral rivaroxaban for symptomatic venous thromboembolism. N Engl J Med. 2011;364(12):1178. 10.1056/NEJMc1100734 21428778

[10] Büller HR, Prins MH, Lensing AWA (2012). Oral rivaroxaban for the treatment of symptomatic pulmonary embolism. N Engl J Med.

[11] Posch F, Königsbrügge O, Zielinski C (2015). Treatment of venous thromboembolism in patients with cancer: a network meta-analysis comparing efficacy and safety of anticoagulants. Thromb Res.

[12] van Es N, Di Nisio M, Bleker SM (2015). Edoxaban for treatment of venous thromboembolism in patients with cancer: rationale and design of the hokusai VTE-cancer study. Thromb Haemost.

[13] Young AM, Marshall A, Thirlwall J (2018). Comparison of an oral factor xa inhibitor with low molecular weight heparin in patients with cancer with venous thromboembolism: results of a randomized trial (SELECT-D). J Clin Oncol.

[14] Streiff MB, Holmstrom B, Angelini D (2018). NCCN Guidelines® insights cancer-associated venous thromboembolic disease, version 2.2018 featured updates to the NCCN guidelines. J Natl Compr Cancer Netw.

[15] Key NS, Khorana AA, Kuderer NM, et al. Venous thromboembolism prophylaxis and treatment in patients with Cancer: ASCO clinical practice guideline update. J Clin Oncol. 2019;38(5):496–520. 10.1200/JCO.19.01461 31381464

[16] Chaudhury A, Balakrishnan A, Thai C (2018). The efficacy and safety of rivaroxaban and Dalteparin in the treatment of Cancer associated venous thrombosis.

[17] Horsted Freesia, West Joe, Grainge Matthew J. (2012). Risk of Venous Thromboembolism in Patients with Cancer: A Systematic Review and Meta-Analysis. PLoS Medicine.

[18] Lee EC, Cameron SJ (2017). Cancer and thrombotic risk: the platelet paradigm.

[19] Schulman S, Kakkar AK, Goldhaber SZ (2014). Treatment of acute venous thromboembolism with dabigatran or warfarin and pooled analysis. Circulation.

[20] DeHaas KA (2017). The direct Oral anticoagulants Apixaban, rivaroxaban, and edoxaban.

[21] Eriksson BI, Quinlan DJ, Eikelboom JW (2011). Novel Oral Factor Xa and Thrombin Inhibitors in the Management of Thromboembolism. Annu Rev Med.

